# Numerical assessment of irreversibility in radiated Sutterby nanofluid flow with activation energy and Darcy Forchheimer

**DOI:** 10.1038/s41598-023-46439-8

**Published:** 2023-11-03

**Authors:** Mujeeb ur Rahman, Fazal Haq, M. Ijaz Khan, Fuad A. Awwad, Emad A. A. Ismail

**Affiliations:** 1 Department of Mathematical Sciences, Karakoram International University, Gilgit, 15100 Pakistan; 2 Department of Mathematics, Riphah International University, Islamabad Campus, Islamabad, 44000 Pakistan; 3 Department of Mechanical Engineering, Lebanese American University, Kraytem, Beirut, 1102-2801 Lebanon; 4 Department of Mechanics and Engineering Science, Peking University, Beijing 100871, China; 5 Department of Quantitative Analysis, College of Business Administration, King Saud University, P.O. Box 71115, 11587 Riyadh, Saudi Arabia

**Keywords:** Biophysics, Mathematics and computing, Nanoscience and technology

## Abstract

Entropy generation is a concept that is primarily associated with thermodynamics and engineering, and it plays a crucial role in understanding and optimizing various processes and systems. Applications of entropy generation can be seen in turbo machinery, reactors, chillers, desert coolers, vehicle engines, air conditioners, heat transfer devices and combustion. Due to industrial applications entropy generation has gained attention of researchers. Owing such applications, current communication aims to model and analyzed the irreversibility in Sutterby nanoliquid flow by stretched cylinder. Momentum equation is reported by considering porosity, Darcy Forchheimer and magnetic field. While in energy equation radiation and Joule heating effects are accounted. Activation energy impact is accounted in the modeling of concentration equation. Thermodynamics second law is utilized for physical description of irreversibility analysis. Through similarity transformations dimensional equations representing flow are transformed to dimensionless ones. Numerical solution for ordinary system is obtained via Runge–Kutta-Fehlberg scheme in Mathematica platform through NDsolve code. Influence of prominent variables on velocity, entropy, temperature, Bejan number and concentration are graphically analyzed. Coefficient of skin friction, gradient of temperature and Sherwood number are numerically analyzed. The obtained results show that velocity field decreases through higher porosity and Forchheimer variables. Velocity and temperature curves shows an opposite trend versus magnetic parameter. A decay in concentration distribution is noticed through larger Schmidt number. Entropy generation amplifies against magnetic parameter and Brinkman number.

## Introduction

Viscoelastic liquids like molten polymers and polymer solutions exhibit numerous verities of rheological characteristics. These features include time dependent viscosity, shear rate dependent viscosity, normal stresses in steady shear flow and various time-dependent elastic effects. Sutterby fluid model is one of the most important models proposed by Sutterby^1^, which addresses the solution of high polymer aqueous solutions. Imran et al.^2^ inspected the thermal radiation and chemical reaction influences on Sutterby nanomaterial flow in inclined elastic channel. Impact of radiation, mixed convection and Joule heating on Sutterby nanoliquid flow in a vertical channel is numerically examined by Hayat et al.^3^. Akbar and Nadeem^4^ reported mixed convection flow of Sutterby nanoliquid in a diverging tube. Helical flow of Sutterby nanomaterial between two concentric cylinders is scrutinized by Batra and Eissa^5^. Ishtiaq et al.^6^ examined shear thickening/thinning behavior of Sutterby nanomaterial flow over biaxially stretchable sheet with magnetic field and heat source/sink. Mixed convection and Arrhenius kinetics effects on 3-D steady flow of Sutterby nanomaterial is conveyed by Azam et al.^7^ .Khan et al.^8^ inspected the features of stratified Sutterby nanomaterial flow in presence of external radiation and Lorentz force.

Mixture of nano-sized metallic particles and base fluids are nanofluids/nanomaterials. Nano-sized metallic particles include metallic oxides, metals, carbon nanotubes and nitrides. Conventional base fluids are water, ethylene glycol and light oils. Nanofluids have higher thermal performance as compared to conventional carrier liquids. Practical usages of such nanofluids can be seen in thermal engineering processes like fuel cells, refrigerators and engine oil. Firstly Choi^9^ added nano-sized metallic particles in carrier fluids and concluded that thermal features improved significantly. Buongiorno^10^ provided a model to study heat transfer augmentation in nanomaterials. He considered seven slip mechanisms for nanoparticles and proved that thermophoresis diffusion and Brownian movement are governing factors as compared to others. Prasad et al.^11^ reported radiative nanofluid flow with Lorentz force effect. Turkyilmazoglu^12^ inspected mass and heat transportation in nanofluid flow over different frames using Buongiorno model. Tian et al.^13^ analyzed convectively heated MHD nanoliquid flow having stagnation point over stretching sheet. Features of laminar flow of viscous fluid flow by stretchable cylinder are analytically examined by Turkyilmazoglu^14^. Hayat et al.^15^ analyzed convective hybrid nanofluid flow with radiation and heat transfer characteristics. Influences of Hall current and electrical MHD in flow of micropolar nanofluid between a pair of rotating disks is explored by Awan et al.^16^. Hussain et al.^17^ inspected features of rotating flow of hybrid nanoliquid accounting the influences of restricted slip boundary constraints. Qureshi et al.^18^ examined impressions of heat generation and magnetic field in hybrid peristaltic flow in a metachronal wave. Parveen et al.^19^ inspected the characteristics of dissipative bioconvective flow of nanoliquid which contains chemotactic microorganisms through Joule heating. Rheological properties of Pseudo plastic nanomaterial flow in a symmetric channel accounting the effects of ciliary motion is presented by Khan et al.^20^. Bayesian regularization networks is implemented to explore the features of nanofluid flows considering various effects by Awan et al.^21,22^. Ahmad et al.^23^ deliberated Maxwell nanomaterial flow by permeable rotating frame with heat transfer analysis. Flow behavior of radiative MHD Maxwell nanoliquid considering viscous dissipation effects is investigated by Hsiao^24^. Hassan et al.^25^ inspected the behavior of MHD hybrid nanomaterial flow which contains SWCNT-Ag as nanoparticles with radiation effects. A few more studies related to nanofluid are given in refs.^26–30^.

Due to applications in environmental, chemical, industrial and pharmaceutical sectors nanoliquid flows over porous surface have gained significance. Applications of such flows may include in energy storage units, geothermal heat exchanger layouts, nuclear waste disposal and crude oil production. Seddeek^31^ reported convective nanofluid flow having thermophoresis and dissipation features saturating porous space. Umavathi et al.^32^ described Darcy-Forchheimer convective nanofluid flow with Brinkman relation. Muhammad et al.^33^ analyzed Maxwell nanoliquid flow in Dacry-Forchheimer porous space. Hayat et al.^34^ scrutinized Darcy-Forchheimer flow with carbon nanotubes in presence of permeability. Alzahrani^35^ investigated bidirectional flow of CNTs in Darcy-Forchheimer and porous surface. Turkyilmazoglu^36^ studied the features magnetic field of uniform strength applied horizontally to the flow generated by rotating disk. Hayat et al.^37^ discovered Carreau nanoliquid flow due to heated surface with Darcy-Forchheimer and porosity effects.

In chemical reactions role of activation energy (AE) is noteworthy. The lowest energy amount obligatory to trigger a chemical reaction is named as AE. In different fields AE concept is widely used such as water mechanics, oil emulsions and oil tank counting. Bestman^38^ revealed flow over porous surface in existence of energy activation and binary response with mass and heat transmission characteristics. Makinde et al.^39^ reported steady radiative flow over porous medium with heat transfer and chemical reaction impacts in an optically reedy atmosphere. Maleque^40^ discovered unsteady boundary layer flow having heat sink/source and energy activation impacts. Awad et al.^41^ investigated unsteady rotational viscid fluid flow considering AE and chemical reaction. Impacts of AE and thermal radiation on MHD flow of Carreau are reported by Hsiao^42^.

Entropy generation (EG) is an extensive property of thermodynamics. Thermodynamics second law states that in an isolated thermal system entropy never decays. In an irreversible reaction total entropy always increases while in reversible reactions it remains constant. Bejan^43^ studied irreversibility in convective nanoliquid flow. Turkyilmazoglu^44^ scrutinized EG and slip impressions in radiated fluid flow in by metallic permeable channel. Khan et al.^45^ calculated the EG in radiative flow of Sisko liquid by stretched surface with dissipation effect. Vatanmakan et al.^46^ examined EG in steam flow with volumetric heating and turbine blades. Hayat et al.^47^ inspected EG in Casson type nanoliquid flow by stretchable sheet through magnetic field and Arrhenius kinetics. Gul et al.^48^ reported EG in viscoelastic Poiseuille nanofluid flow. Xie and Jian^49^ discussed entropy optimization rate in MHD nanofluid flow through micro parallel networks. Khan et al.^50^ scrutinized irreversibility in rotational viscous nanofluid flow with mixed convection and radiative heat flux. Huminic and Huminic^51^ reported irreversibility in hybrid nanomaterials flow.

The literature cited in above transpires that irreversibility in Darcy Forchheimer flow of Sutterby nanomaterial due to stretched cylinder with porous walls in existence of Arrhenius kinetics, Joule heating and chemical reaction is not examined till now. In order to fill this gape, motivation here is to investigate the irreversibility in radiative Sutterby nanoliquid flow by stretchable cylinder. Heat transport characteristics are scrutinized through Joule hating and radiation effects. Furthermore, Brownian movement and thermophoresis diffusion impacts are accounted. Mass transfer characteristics are reported through activation energy. Utilizing thermodynamics second law physical description of irreversibility is analyzed.

## Problem description

**Figure 1:**
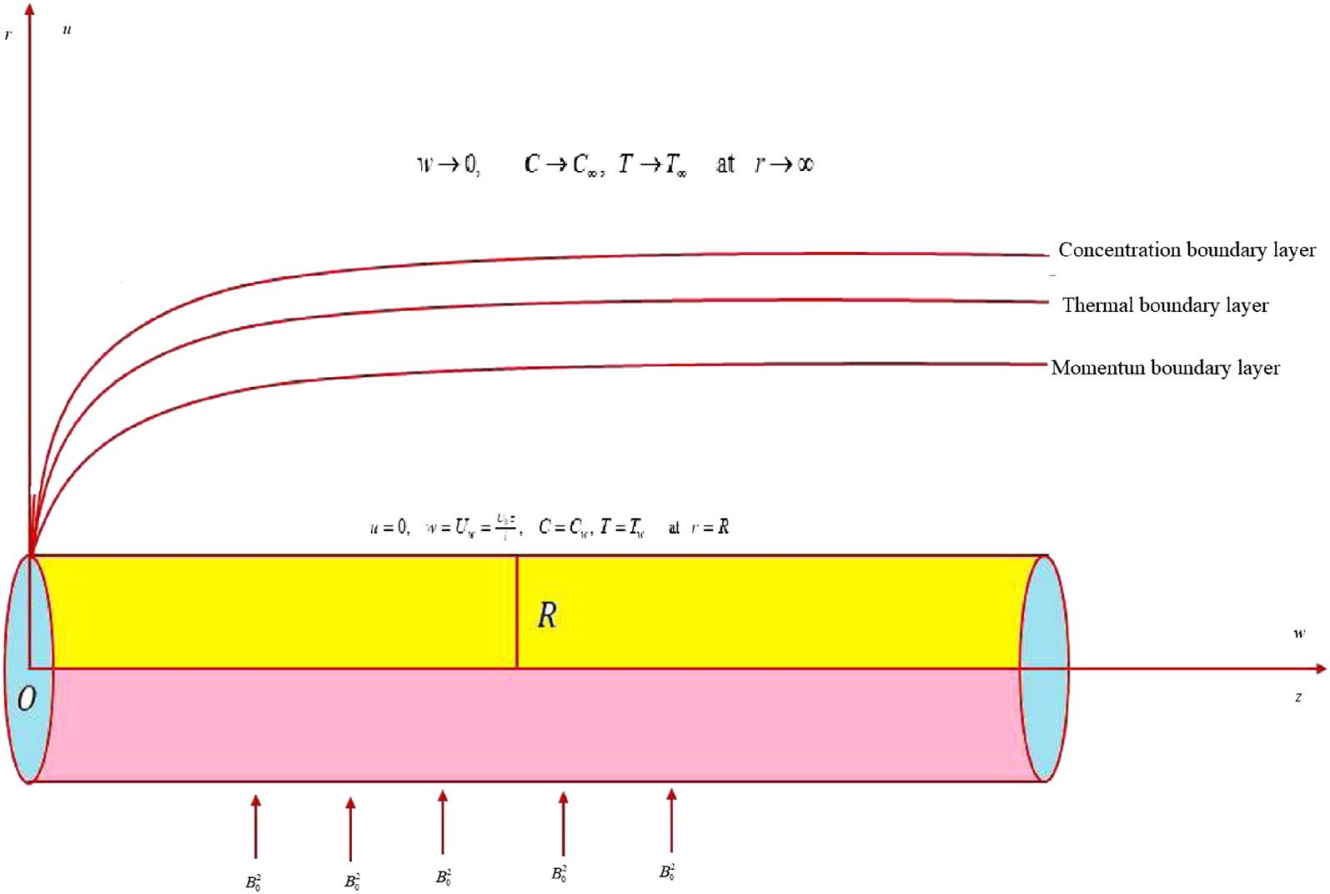
Coordinate system and flow diagram.

In current inspection, incompressible steady flow of Sutterby nanomaterial by stretchable cylinder is considered. The flow is taken along axial *z*-direction and radial direction is taken normal to *z*-direction. The cylinder stretches with velocity $U_{w} = \tfrac{{U_{0} z}}{l}$ in the axial direction due which flow generates. Magnetic field of constant strength $B_{0}$ is imposed vertical to the flow. Induced magnetic field effect is neglected for small Reynolds number. Effects of Arrhenius kinetics, Joule heating and radiation have been incorporated in thermal and mass concentration equations. Boundary layer conventions are accounted in development of flow governing model equations. Schematic flow diagram with boundary restrictions is depicted in Fig. 1. The governing equations signifying the flow under above norms are as follows^52,53^;

1 $$\frac{\partial u}{{\partial r}} + \frac{u}{r} + \frac{\partial w}{{\partial z}} = 0,$$

2 $$u\frac{\partial w}{{\partial r}} + w\frac{\partial w}{{\partial z}} = \frac{\nu }{2r}\frac{\partial w}{{\partial r}} + \frac{\nu }{2}\frac{{\partial^{2} w}}{{\partial r^{2} }} - \frac{{\nu mB^{2} }}{4}\left( {\frac{\partial w}{{\partial r}}} \right)^{2} \frac{{\partial^{2} w}}{{\partial r^{2} }} - \frac{{\sigma B_{0}^{2} }}{\rho }w - \frac{\mu }{{\rho k_{p} }}w - F_{e} w^{2} ,$$

3 $$u\tfrac{\partial T}{{\partial r}} + w\tfrac{\partial T}{{\partial z}} = \tfrac{k}{{\rho C_{p} }}\left( {\tfrac{{\partial^{2} T}}{{\partial r^{2} }} + \tfrac{1}{r}\tfrac{\partial T}{{\partial r}}} \right) + \tau \left[ {D_{B} \tfrac{\partial C}{{\partial r}}\tfrac{\partial T}{{\partial r}} + \tfrac{{D_{T} }}{{T_{\infty } }}\left( {\tfrac{\partial T}{{\partial r}}} \right)^{2} } \right] + \tfrac{{\sigma B_{0}^{2} }}{{\rho C_{p} }}w^{2} + \tfrac{1}{{\rho C_{p} }}\tfrac{{16\sigma^{ * } T_{\infty }^{3} }}{{3k^{ * } }}\tfrac{{\partial^{2} T}}{{\partial r^{2} }},$$

4 $$u\tfrac{\partial C}{{\partial r}} + w\tfrac{\partial C}{{\partial z}} = D_{B} \left( {\tfrac{{\partial^{2} C}}{{\partial r^{2} }} + \tfrac{1}{r}\tfrac{\partial C}{{\partial r}}} \right) + \tfrac{{D_{T} }}{{T_{\infty } }}\left( {\tfrac{{\partial^{2} T}}{{\partial r^{2} }} + \tfrac{1}{r}\tfrac{\partial T}{{\partial r}}} \right) - k_{r}^{2} \left( {C - C_{\infty } } \right)\left( {\tfrac{T}{{T_{\infty } }}} \right)^{{n_{1} }} \exp \left( {\tfrac{ - Ea}{{kT}}} \right),$$

with

5 $$\left. {\begin{array}{*{20}c} {u = 0, \, w = U_{w} = \tfrac{{U_{0} \,z}}{l}, \, T = T_{w} ,\,\,\,C = C_{w} \,\,\,at \, \,\,r = R} \\ {w \to 0, \, C \to C_{\infty } ,\,\,\,\,T \to T_{\infty } \,\,\, \, at \, r \to \infty ,} \\ \end{array} } \right\}.$$

Considering^53^

6 $$\left. {u = - \sqrt {\tfrac{{\nu U_{0} }}{l}} \tfrac{R}{r}f\left( \eta \right),w = \tfrac{{U_{0} z}}{l}f^{\prime}\left( \eta \right),\eta = \sqrt {\tfrac{{U_{0} }}{\nu l}} \left( {\tfrac{{r^{2} - R^{2} }}{2R}} \right),\,\theta \left( \eta \right) = \tfrac{{T - T_{\infty } }}{{T_{w} - T_{\infty } }}, \, \phi \left( \eta \right) = \tfrac{{C - C_{\infty } }}{{C_{w} - C_{\infty } }}} \right\}.$$

One has

7 $$\left. \begin{aligned} \left( {1 + 2\gamma_{3} \eta } \right)f^{\prime\prime\prime} + 2\gamma_{3} f^{\prime\prime} - 2\beta_{1} Re\gamma_{3} \left( {1 + 2\gamma_{3} \eta } \right)f^{{\prime \prime^{2} }} - 2\beta_{1} Re\left( {1 + 2\gamma_{3} \eta } \right)^{2} f^{{\prime \prime^{2} }} f^{\prime\prime\prime} \hfill \\ - 2\lambda f^{\prime} - 2Mf^{\prime} - 2Frf^{{\prime^{2} }} - 2f^{{\prime^{2} }} + 2ff^{\prime\prime} = 0 \hfill \\ \end{aligned} \right\},$$

8 $$\left. \begin{aligned} \left( {1 + 2\gamma_{3} \eta } \right)\theta^{\prime\prime} + \Pr f\theta^{\prime} + 2\gamma_{3} \theta^{\prime} + \left( {1 + 2\gamma_{3} \eta } \right)\Pr Nb\theta^{\prime}\phi^{\prime} + \left( {1 + 2\gamma_{3} \eta } \right)\Pr Nt\theta^{{\prime^{2} }} \hfill \\ + \Pr MEcf^{{\prime^{2} }} + Rd\left[ {\gamma_{3} \theta^{\prime} + \left( {1 + 2\gamma_{3} \eta } \right)\theta^{\prime\prime}} \right] = 0 \hfill \\ \end{aligned} \right\},$$

9 $$\left. \begin{aligned} \left( {1 + 2\gamma_{3} \eta } \right)\phi^{\prime\prime} + \gamma_{3} \phi^{\prime} + \tfrac{Nt}{{Nb}}\left[ {\left( {1 + 2\gamma_{3} \eta } \right)\theta^{\prime\prime} + \gamma_{3} \theta^{\prime}} \right] + Scf\phi^{\prime} \hfill \\ - Sc\gamma \phi \left( {1 + \delta_{1} \theta } \right)^{n1} \exp \left( {\tfrac{{ - E_{1} }}{{1 + \delta_{1} \theta }}} \right) = 0 \hfill \\ \end{aligned} \right\},$$

with

10 $$\left. {\begin{array}{*{20}c} {f\left( 0 \right) = 0,\theta \left( 0 \right) = 1, \, \phi \left( 0 \right) = 1,\,\, \, f^{\prime}\left( 0 \right) = 1,} \\ {f^{\prime}\left( \infty \right) = 0, \, \theta \left( \infty \right) = 0, \, \phi \left( \infty \right) = 0.} \\ \end{array} } \right\}$$

Here

11 $$\left. {\begin{array}{*{20}c} {\begin{array}{*{20}c} {\gamma_{3} = \sqrt {\tfrac{\nu l}{{U_{0} R^{2} }}} , \, {\text{Re}} = \tfrac{{U_{0} z^{2} }}{\nu l}, \, M = \sqrt {\tfrac{{\sigma B_{0}^{2} l}}{{\rho U_{0} }}} , \, \lambda = \tfrac{\nu l}{{k_{p} U_{0} }}, \, \beta_{1} = \tfrac{{mB^{2} U_{0}^{2} }}{{4l^{2} }}, \, \Pr = \tfrac{{\mu c_{p} }}{k},} \\ {Fr = \tfrac{{C_{b} }}{{\sqrt {k_{p} } }}, \, Rd = \tfrac{{4\sigma^{ * } T_{\infty }^{3} }}{{kk^{ * } }},Nb = \tfrac{{\tau D_{B} \left( {C_{w} - C_{\infty } } \right)}}{\upsilon },Nt = \tfrac{{\tau D_{T} \left( {T_{w} - T_{\infty } } \right)}}{{T_{\infty } \upsilon }},} \\ \end{array} } \\ {\delta_{1} = \tfrac{{T_{w} - T_{\infty } }}{{T_{\infty } }} \, ,Ec = \tfrac{{U_{w}^{2} }}{{C_{p} \left( {T_{w} - T_{\infty } } \right)}}, \, E_{1} = \tfrac{{E_{a} }}{{kT_{\infty } }}, \, \gamma = \tfrac{{k_{r}^{2} l}}{{U_{0} }}, \, Sc = \tfrac{\nu }{{D_{B} }}} \\ \end{array} } \right\}.$$

### Physical quantities

Physical quantities are as follows;

12 $$\left. {Cf_{z} = \tfrac{{ - \left( {\tau_{w} } \right)_{r = R} }}{{\rho u_{w}^{2} }},\;Nu_{z} = \tfrac{{zq_{w} }}{{k\left( {T_{w} - T_{\infty } } \right)}},\,\,\,Sh_{z} = \tfrac{{zq_{m} }}{{D_{B} \left( {C_{w} - C_{\infty } } \right)}}.} \right\}$$

where

13 $$\left. {q_{w} = - k\left( {\frac{\partial T}{{\partial y}} + \frac{{16\sigma^{ * } T_{\infty }^{3} }}{{kk^{ * } }}} \right)\left( {\frac{\partial T}{{\partial r}}} \right)_{r = R} , \, q_{m} = - D_{B} \left( {\frac{\partial C}{{\partial r}}} \right)_{r = R} .} \right\}$$

Final forms are

14 $$\begin{aligned} \left. {Cf_{z} \left( {{\text{Re}}_{z} } \right)^{{\tfrac{1}{2}}} = - f^{\prime\prime}\left( 0 \right) - \tfrac{{\beta_{1} }}{4}f^{\prime\prime}(0)\left[ {4f^{\prime}(0) + 4\gamma_{3}^{2} f(0)} \right]^{2} - 4f(0)f^{\prime}(0) + \lambda \left[ {f^{\prime\prime}(0)} \right]^{2} } \right\} \hfill \\ Nu_{z} \left( {{\text{Re}}_{z} } \right)^{{ - \tfrac{1}{2}}} = - \left[ {1 + Rd} \right]\theta^{\prime}\left( 0 \right), \, Sh_{z} \left( {{\text{Re}}_{z} } \right)^{{ - \tfrac{1}{2}}} = - \phi^{\prime}\left( o \right), \hfill \\ \end{aligned}$$

where $Re_{z} \left( { = \tfrac{{zU_{w} }}{\nu }} \right)$ is local Reynolds number.

### Entropy modeling

It is defined as^52,53^;

15 $$E_{G} = \tfrac{k}{{T_{\infty }^{2} }}\left( {1 + Rd} \right)\left( {\tfrac{\partial T}{{\partial r}}} \right)^{2} + \tfrac{\mu }{{T_{\infty } k_{p} }}w^{2} + \tfrac{RD}{{T_{\infty } }}\left( {\tfrac{\partial T}{{\partial r}}} \right)\left( {\tfrac{\partial C}{{\partial r}}} \right) + \tfrac{{\sigma B_{0}^{2} }}{{T_{\infty } }}w^{2} + \tfrac{RD}{{C_{\infty } }}\left( {\tfrac{\partial C}{{\partial r}}} \right)^{2} ,$$

non-dimensional form of entropy generation rate is

16 $$\left. \begin{aligned} S_{G} = \tfrac{{E_{G} }}{{E_{{G_{0} }} }} & = \left( {1 + Rd} \right)\left[ {1 + 2\gamma _{3} \eta } \right]\delta _{1} \theta ^{{\prime 2}} + MB_{r} f^{{\prime 2}} + \left( {1 + 2\gamma _{3} \eta } \right)L\theta ^{\prime}\phi ^{\prime} \\ & \;\;\,\,\, + \left( {1 + 2\gamma _{3} \eta } \right)L\tfrac{{\alpha _{2} }}{{\delta _{1} }}\phi ^{{\prime 2}} + B_{r} \lambda f^{{\prime 2}} , \\ \end{aligned} \right\}$$

Bejan number is

17 $$\left. {Be = \frac{{\left( {1 + Rd} \right)\left[ {1 + 2\gamma_{3} \eta } \right]\delta_{1} \theta^{\prime 2} + \left( {1 + 2\gamma_{3} \eta } \right)L\theta^{\prime}\phi^{\prime}}}{{\begin{array}{*{20}c} {\left( {1 + Rd} \right)\left[ {1 + 2\gamma_{3} \eta } \right]\delta_{1} \theta^{\prime 2} + MB_{r} f^{\prime 2} + \left( {1 + 2\gamma_{3} \eta } \right)L\theta^{\prime}\phi^{\prime}} \\ { + \left( {1 + 2\gamma_{3} \eta } \right)L\tfrac{{\alpha_{2} }}{{\delta_{1} }}\phi^{\prime 2} + B_{r} \lambda f^{\prime 2} ,} \\ \end{array} }}} \right\}$$

here $$S_{G}$$$$\left( { = \tfrac{{E_{G} T_{\infty } \nu l}}{{U_{0} k\left( {T_{w} - T_{\infty } } \right)}}} \right)$$ denotes the entropy rate, $$E_{{G_{0} }} \left( { = \tfrac{{k\left( {T_{w} - T_{\infty } } \right)}}{{T_{\infty } }}\left( {\tfrac{{U_{0} }}{\nu l}} \right)} \right)$$ the characteristic entropy rate, $$B_{r} \left( { = \tfrac{{\mu U_{0}^{2} z^{2} }}{{l^{2} k\left( {T_{w} - T_{\infty } } \right)}}} \right)$$ Brinkman number, $$L\left( { = \tfrac{{RD\left( {C_{w} - C_{\infty } } \right)}}{k}} \right)$$ diffusion variable and $$\alpha_{2} \left( { = \tfrac{{C_{w} - C_{\infty } }}{{C_{\infty } }}} \right)$$ concentration difference ratio variable.

## Numerical solution and discussion

Here NDsolve code in Mathematica is executed to solve the dimensionless system of equations. Impact of sundry variables on Sutterby nanofluid velocity $(f^{\prime}\left( \eta \right))$, thermal field $\left( {\theta (\eta )} \right)$, mass concentration $\left( {\phi (\eta )} \right)$ EG rate $\left( {S_{G} } \right)$ and Bejan number $\left( {Be} \right)$ are scrutinized by plotting. Engineering quantities are evaluated numerically. Table 1 is labeled to ensure the correctness of present numerical approach. This table demonstrated the comparison of gradients of temperature $\left( {\theta^{^{\prime\prime}} (0)} \right)$ versus different $\Pr$ values while influence of remaining variables is neglected. Clearly the results are in good agreement.

**Table 1:** Comparison of results for gradient of temperature.

$\Pr$	Present results	Wang^54^	Gorla and Sidawi^55^
0.07	0.0645	0.0656	0.06562
0.20	0.1689	0.1691	0.1691
0.70	0.4538	0.4539	0.53488
2.00	0.9113	0.9114	0.91142
7.00	1.8953	1.8954	1.89046
20.00	3.3539	3.3539	3.35391

### Velocity

**Figure 2:**
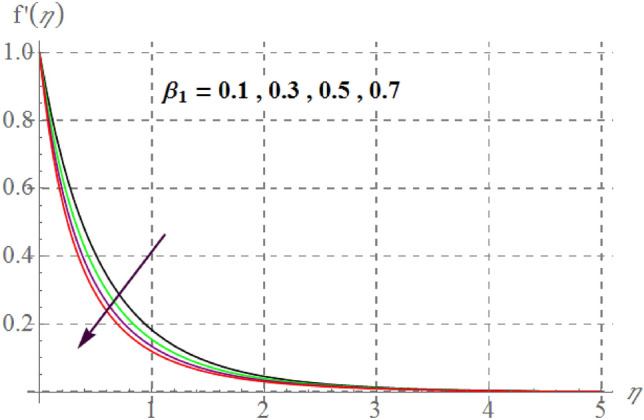
$$f^{\prime}(\eta )$$ curves versus $$\beta_{1}$$.

**Figure 3:**
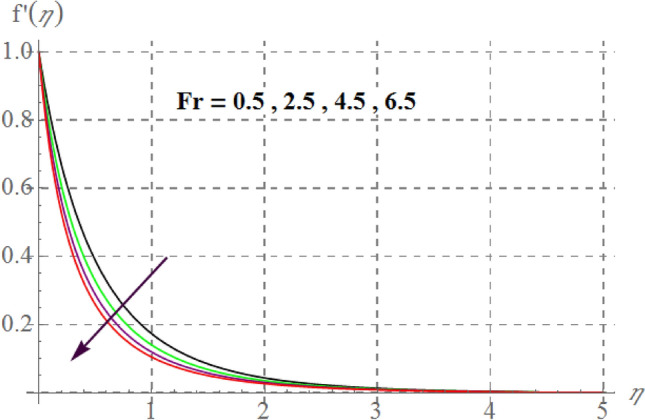
$$f^{\prime}(\eta )$$ curves via $$Fr$$.

**Figure 4:**
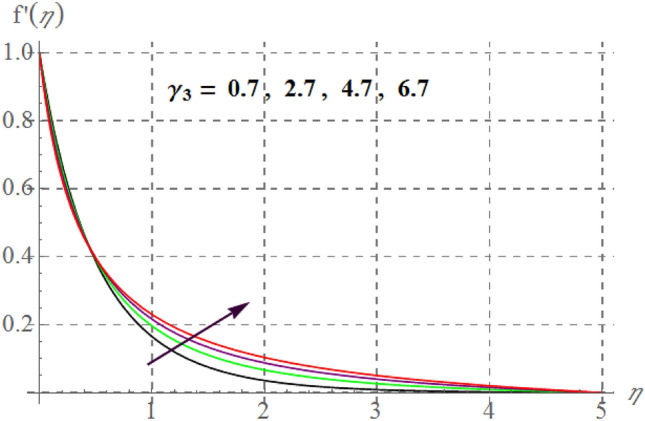
$$f^{\prime}(\eta )$$ curves through $$\gamma_{3}$$.

**Figure 5:**
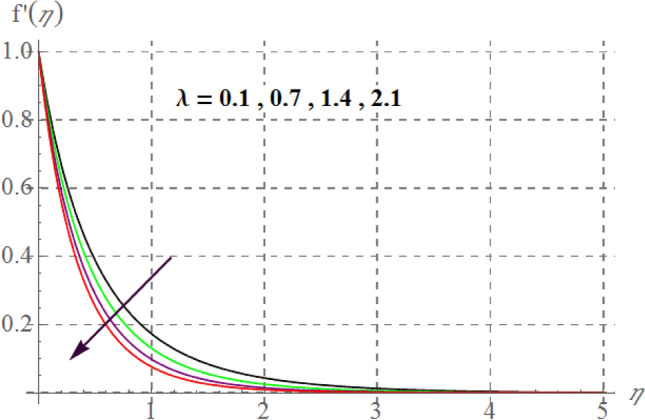
$$f^{\prime}(\eta )$$ curves against $$\lambda$$.

**Figure 6:**
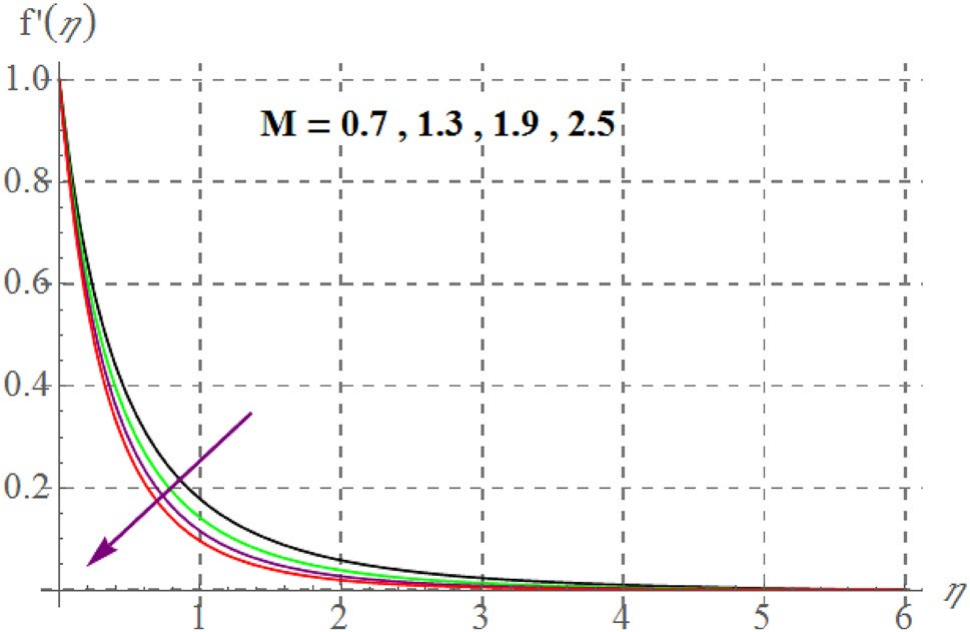
$$f^{\prime}(\eta )$$ curves versus $$M$$.

**Figure 7:**
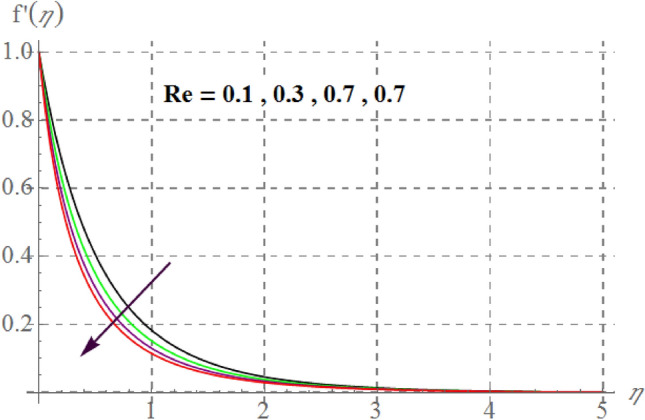
$$f^{\prime}(\eta )$$ curves via $${\text{Re}}$$.

Figure 2 delineates Sutterby fluid parameter $\left( {\beta_{1} } \right)$ impact on $f^{\prime}(\eta )$. For raising values of $\beta_{1}$ velocity decreases. Physically for raising $\beta_{1}$ relaxation time increases as a result viscous effects dominants therefore additional resistance is offered to fluid particles thus $f^{\prime}(\eta )$ decreases. Figure 3 discovers influence of Forchheimer variable $\left( {Fr} \right)$ on fluid velocity. Physically, for hiking $Fr$ estimations, $f^{\prime}(\eta )$ decreases. For larger values of $Fr$ drag surface force increases, thus $f^{\prime}(\eta )$ decreases. Figure 4 shows influence of $\gamma_{3}$ on $f^{\prime}(\eta ).$ here, material velocity upsurges via rising $\gamma_{3}$. For higher $\gamma_{3}$ cylinder radius decreases as a result fluid contact area between fluid particles and surface of cylinder decreases due to which less resistive force of surface is offered to fluid particles so velocity upsurges. Figure 5 describes influence of surface porosity on $f^{\prime}(\eta )$. Velocity decreases for improvement in $\lambda$. For increasing values of $\lambda$ kinematic viscosity of porous medium upsurges, therefore $f^{\prime}(\eta )$ curves decays. Behavior of $f^{\prime}(\eta )$ versus magnetic variable is captured in Fig. 6. Velocity diminishes versus higher $M$. Since through higher $M$ Lorentz force enhances which acts in the opposite direction of flow and thus velocity decays. From Fig. 7 it is noticed that $Re$ and $f^{\prime}(\eta )$ have an inverse relation. In fact $Re$ has direct and inverse relations with inertial forces and viscous forces, thus for larger $Re$ viscous forces dominant over inertial forces thus velocity decreases.

### Temperature

**Figure 8:**
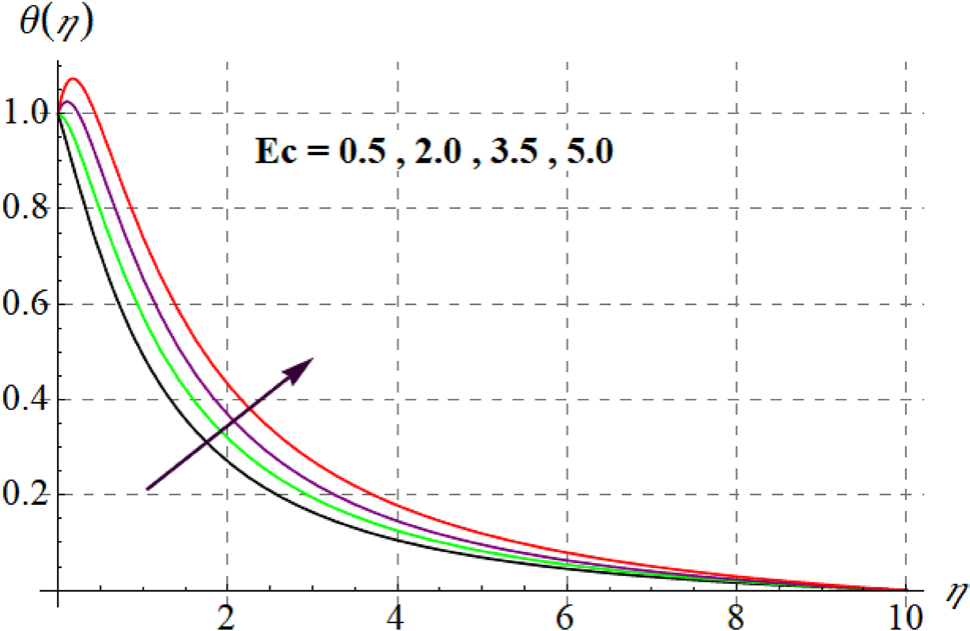
$$\theta (\eta )$$ curves against $$Ec$$.

**Figure 9:**
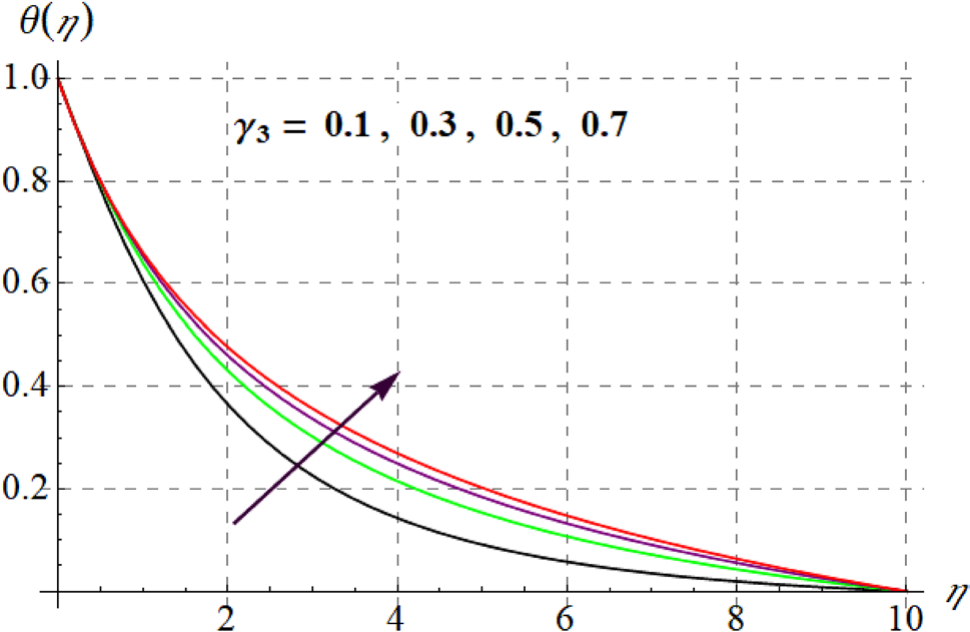
$$\theta (\eta )$$ curves versus $$\gamma_{3}$$.

**Figure 10:**
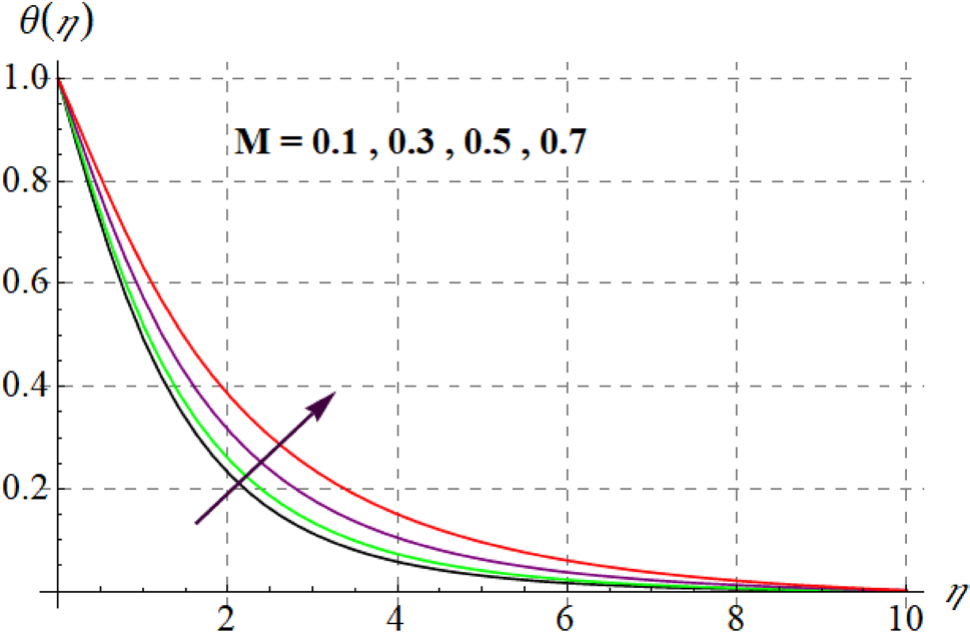
$$\theta (\eta )$$ curves via $$M$$.

**Figure 11:**
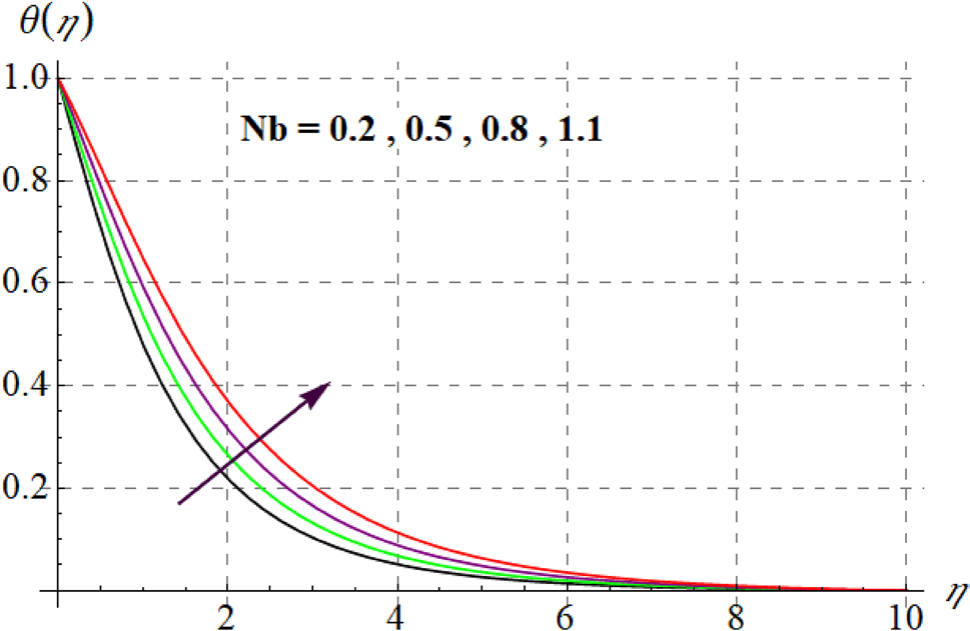
$$\theta (\eta )$$ curves versus $$Nb$$.

**Figure 12:**
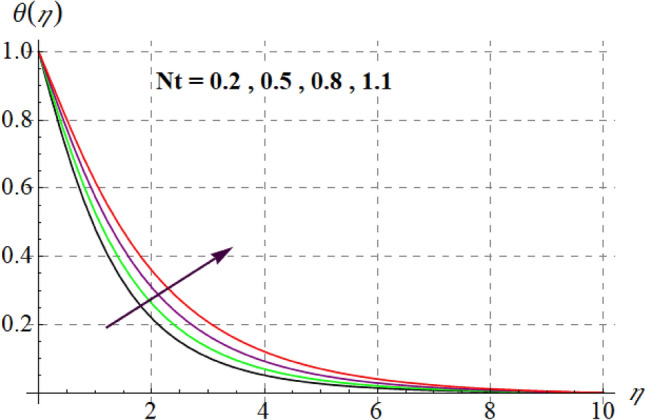
$$\theta (\eta )$$ curves against $$Nt$$.

**Figure 13:**
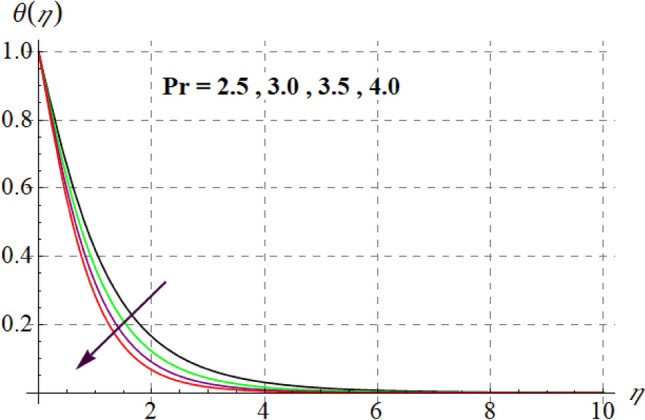
$$\theta (\eta )$$ curves versus $$\Pr$$.

**Figure 14:**
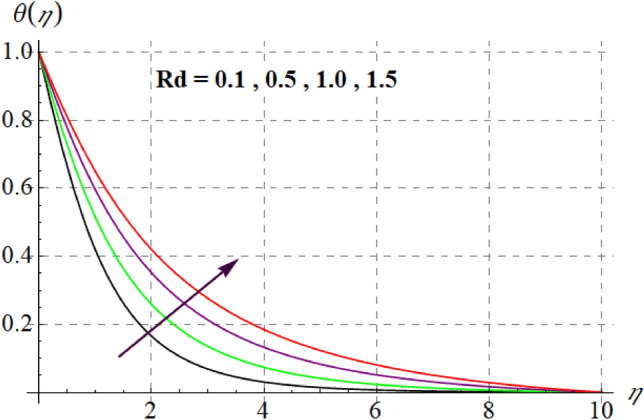
$$\theta (\eta )$$ curves versus $$Rd$$.

Figure 8 gives inspiration of $Ec$ on $\theta (\eta ).$ For enlargement in $Ec$ temperature upturns. Since for higher $Ec$, internal energy of fluid boosts, consequently kinetic energy of system increases as a result inside friction of tiny solid particles enhances and extra heat supplied to the system, resultantly $\theta (\eta )$ increases. Variation in thermal fluid versus $\gamma_{3}$ is captured in Fig. 9. Clearly $\theta (\eta )$ increases for raising values of $\gamma_{3}$. Figure 10 shows the variation in $\theta (\eta )$ against $M$. For greater estimations of $M$ temperature rises. In fact more heat is added in the system when magnetic variable amplifies due to resistive force and so $\theta (\eta )$ increases. Influence of $Nb$ on $\theta (\eta )$ is checked in Fig. 11. Temperature improves through higher $Nb.$ Random movement of tiny particles increases with in fluid against higher $Nb$, consequently inter collision of nanoparticles increases and thus extra heat generates, therefore $\theta (\eta )$ improves. Figure 12 is designed to check the outcome of $Nt$ on $\theta (\eta ).$ For raising $Nt$ thermal field boosts. Physically, when thermophoresis force increases rate of shifting of solid tiny particles from hot to cold region improves, so $\theta (\eta )$ escalates. Figure 13 articulates the influence of $\Pr$ on thermal field. It is perceived here that via higher $\Pr$ thermal field declines. Physically through rising $\Pr$ fluid thermal diffusivity diminished and thus $\theta (\eta )$ retards. Influence of radiation variable on $\theta (\eta )$ is given in Fig. 14. Here $\theta (\eta )$ boosts via larger $Rd.$ since surface heat flux boosts for higher values of $Rd$ and thus $\theta (\eta )$ improves.

### Concentration

**Figure 15:**
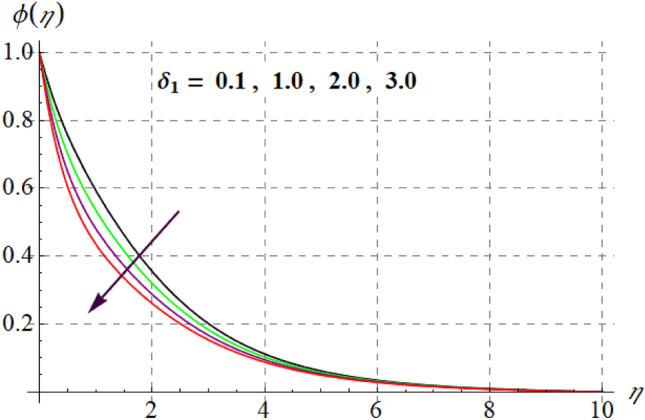
$$\phi (\eta )$$ curves versus $$\delta_{1}$$.

**Figure 16:**
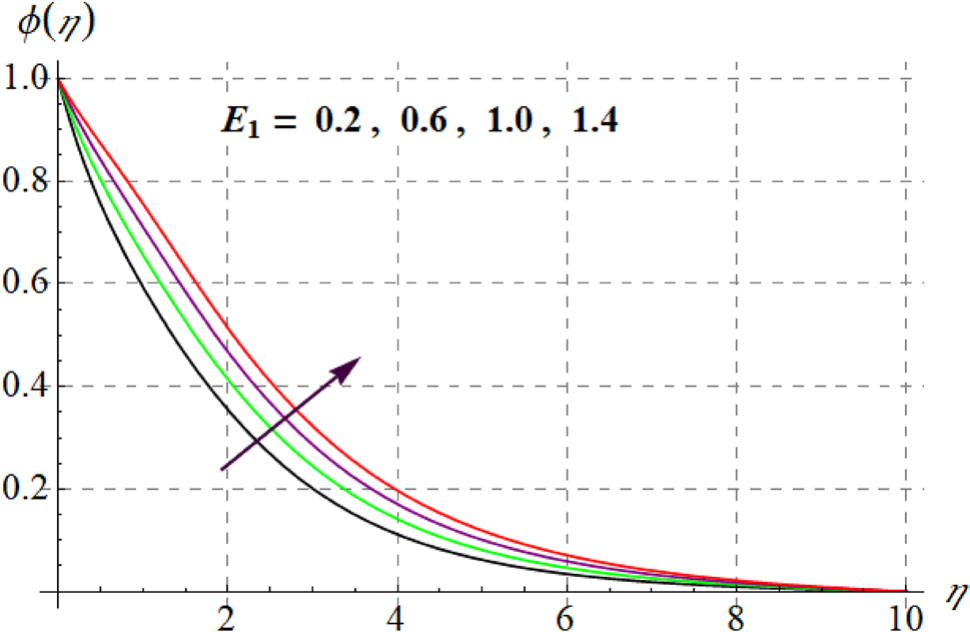
$$\phi (\eta )$$ curves versus $$E_{1}$$.

**Figure 17:**
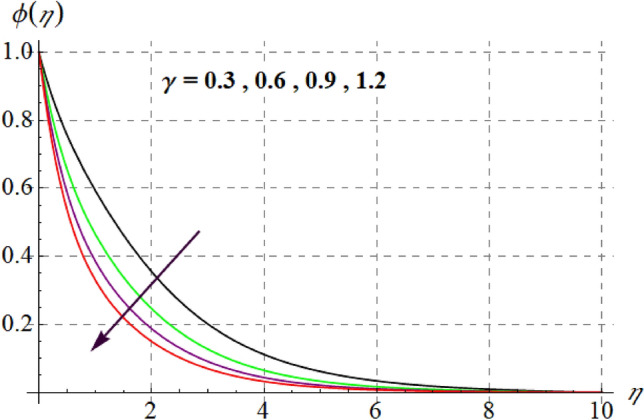
$$\phi (\eta )$$ curves versus $$\gamma$$.

**Figure 18:**
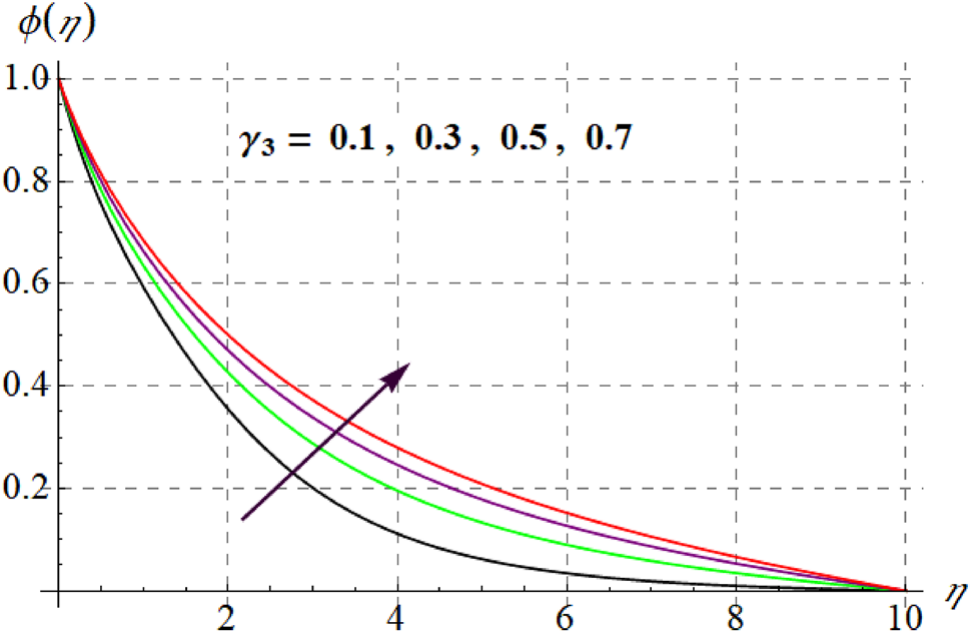
$$\phi (\eta )$$ curves versus $$\gamma_{3}$$.

**Figure 19:**
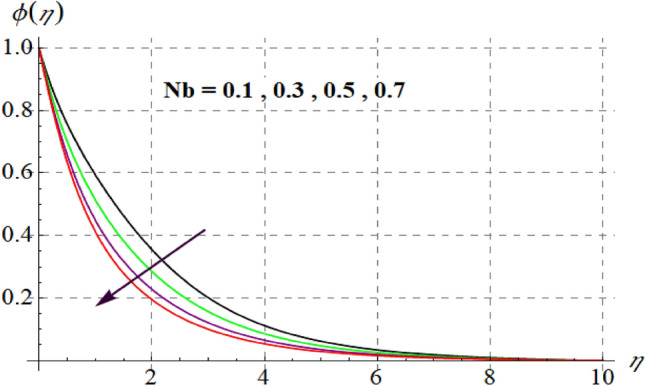
$$\phi (\eta )$$ curves via $$Nb$$.

**Figure 20:**
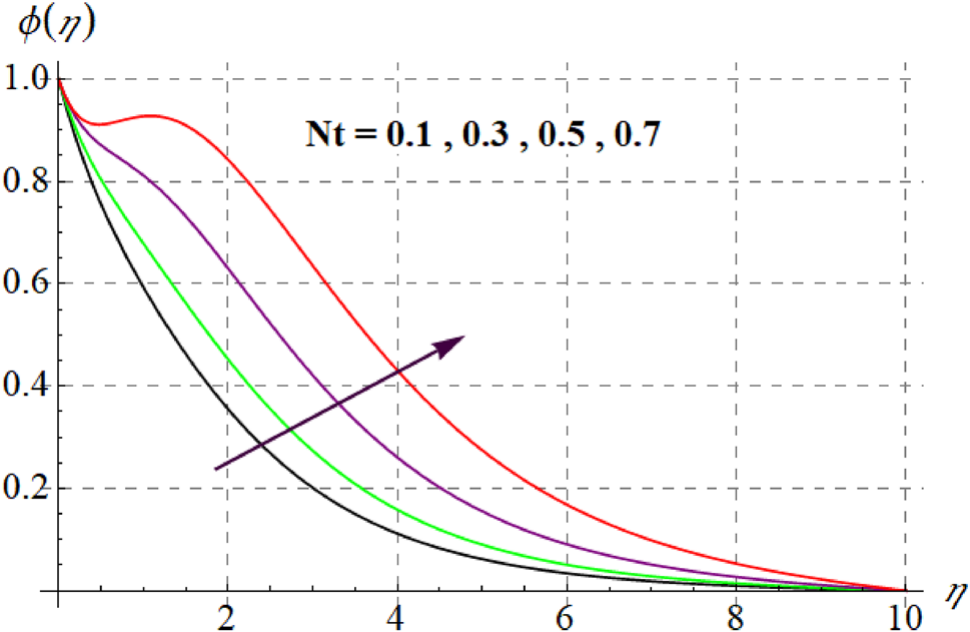
$$\phi (\eta )$$ curves against $$Nt$$.

**Figure 21:**
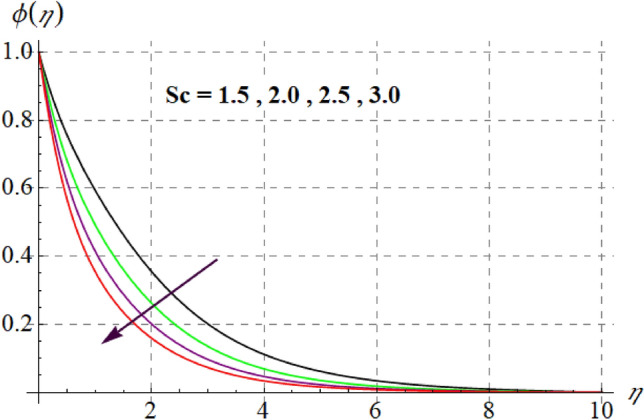
$$\phi (\eta )$$ curves versus $$Sc$$.

Figure 15 is drafted to show influence of $\delta_{1}$ on $\phi (\eta )$. It is observed that for an escalation in $\delta_{1}$ values $\phi (\eta )$ upsurges. Figure 16 depicts $E_{1}$ effect on $\phi (\eta )$ Concentration upsurges for raising values of $E_{1}$. For higher $E_{1}$ Arrhenius function decreases thus concentration increases. Figure 17 portrays effect of $\gamma$ on $\phi (\eta )$. Concentration decreases for raising $\gamma$. For higher $\gamma$ destructive rate of reaction increases and thus liquid species liquefy more successfully, so $\phi (\eta )$ decreases. Figure 18 gives impact of $\gamma_{3}$ on $\phi (\eta )$. For enlargement in $\gamma_{3}$ concentration increases. Figure 19 expresses the $Nb$ influence on $\phi (\eta )$. Nanofluid mass concentration declines through rising $Nb$_._ Physically, intermolecular collision boosts against higher $Nb$ due to arbitrary movement. Resultantly more heat generates thus temperature increases and $\phi (\eta )$ decreases. Figure 20 explores the behavior of $\phi (\eta )$ versus $Nt$. Fluid concentration increases for higher values of $Nt$. Physically, thermophoretic force which shifts fluid particles from hot to cold region increases for enlargement in $Nt$ thus $\phi (\eta )$ increases. Figure 21 is plotted to study influence of $Sc$ on $\phi (\eta )$ Here, $\phi (\eta )$ decreases via increasing values of $Sc.$ Since mass diffusivity reduces for larger Schmidt number and thus $\phi (\eta )$ is diminished.

### Entropy generation and Bejan number

**Figure 22:**
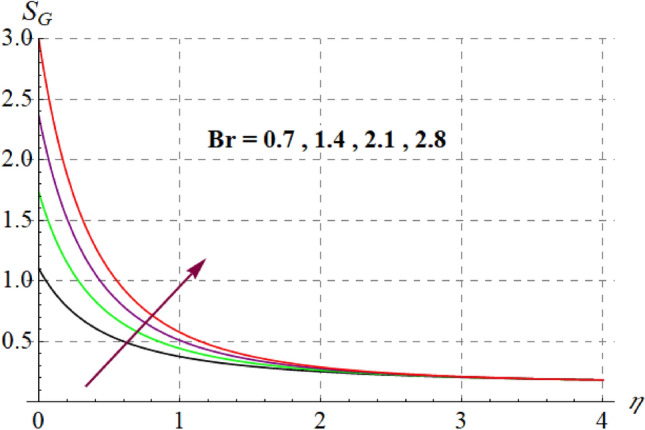
$$S_{G}$$ via $$Br$$.

**Figure 23:**
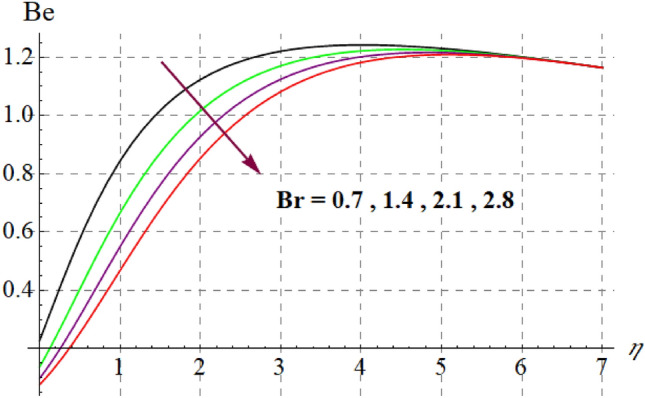
$$Be$$ against $$Br$$.

**Figure 24:**
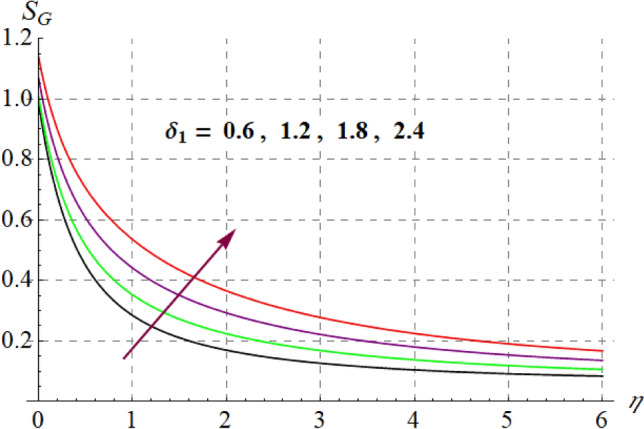
$$S_{G}$$ via $$\delta_{1}$$.

**Figure 25:**
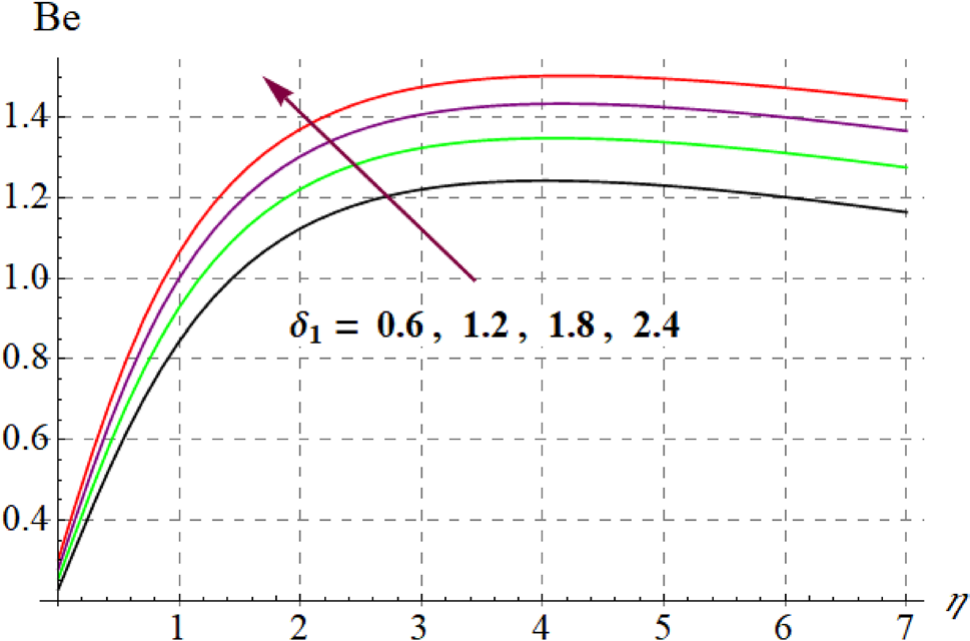
$$Be$$ versus $$\delta_{1}$$.

**Figure 26:**
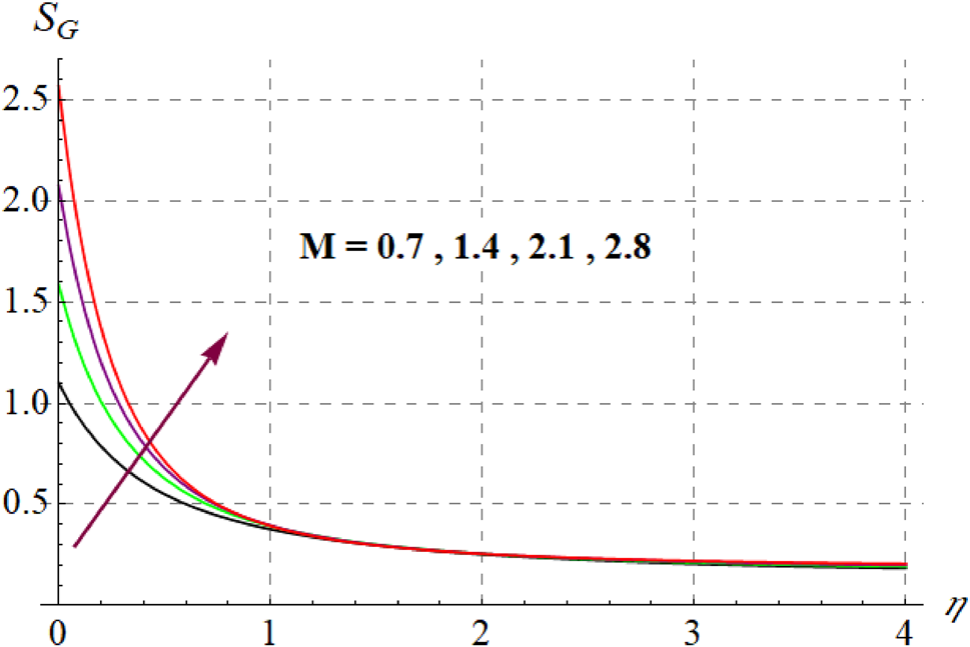
$$S_{G} \,$$ via $$M$$.

**Figure 27:**
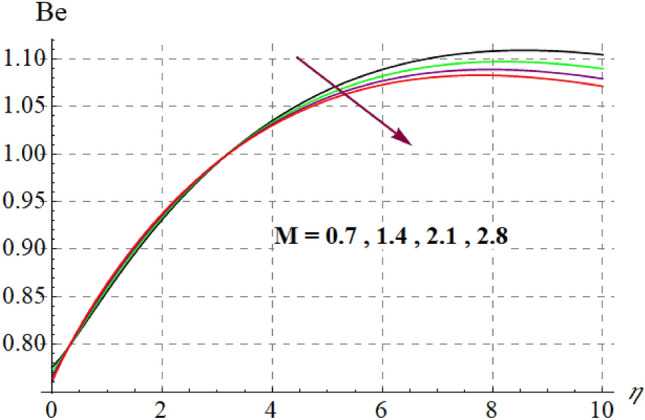
$$Be$$ against $$M$$.

This section is devoted to check the influences of $Br$, $\delta_{1}$ and $M$ on $S_{G}$ and $Be$. Figures 22 and 23 portrays $Br$ influence on $S_{G}$ and $Be$. For higher $Br$ values $S_{G}$ increases while $Be$ decays. Physically, $Br$ acts as heat source within the fluid. Therefore higher $Br$ produces more heat and thus $S_{G}$ enhances whereas $Be$ decays. Figures 24 and 25 shows influence of $\delta_{1}$ on $S_{G}$ and $Be$. For higher $\delta_{1}$ values irreversibility enhances thus both $S_{G}$ and $Be$ increased. Since $\delta_{1}$ is in direct relation with $T_{w} - T_{\infty }$ and we know that $T_{w} > T_{\infty }$, so higher $\delta_{1}$ produces more heat to the fluid and thus both $S_{G}$ and $Be$ enhanced. Outcomes of magnetic variable on $S_{G}$ and $Be$ is expressed in Figs. 26 and 27. It is observed that $S_{G}$ increases for higher $M$ while opposite behavior holds for $Be$. It is due to the fact that $M$ is linked with Lorentz force, which is a resistive force and opposes the flow. Consequently irreversibility with in fluid upsurges and Bejan decays.

### Engineering quantities

Here skin friction coefficient $\left( {Cf_{z} \left( {Re_{z} } \right)^{{\tfrac{1}{2}}} } \right)$, rate of heat transfer $\left( {Nu_{z} \left( {Re_{z} } \right)^{{ - \tfrac{1}{2}}} } \right)$ and Sherwood number $\left( {Sh_{z} \left( {Re_{z} } \right)^{{ - \tfrac{1}{2}}} } \right)$ are discussed numerically. Outcomes of various flow parameters on $Cf_{z} \left( {Re_{z} } \right)^{{\tfrac{1}{2}}}$ are examined in Table 2. For higher $\beta_{1}$, $Fr,$ $\gamma_{3} ,$ $\lambda$, $M$, and $Re$ velocity gradient improves. Computational results of Nusselt number for different flow variables are given in Table 3. Clearly noted that for higher $\Pr$, $M$, $Nt,$ $Nb$ and $Ec$ temperature gradient decreases. An augmentation in $Nu_{z}$ is seen for $Rd$ and $\gamma_{3}$. Variation of various sundry variables on mass transfer rate is examined in Table 4. From this table it is observed that Sherwood number improves for higher $\delta_{1}$, $\gamma ,$ $n,$ $Nb,$ $Nt$ and $Sc$. Opposite effect is seen for $E_{1}$ and $\gamma_{3}$.

**Table 2:** Numerical simulations for $Cf_{z} \left( {Re_{z} } \right)^{{\tfrac{1}{2}}}$.

$\beta_{1}$	$Fr$	$\gamma_{3}$	$\lambda$	$M$	$Re$	$Cf_{z} \left( {Re_{z} } \right)^{{\tfrac{1}{2}}}$
0.6	0.5	0.7	0.2	0.7	0.01	0.232204
0.7						0.487635
0.8						0.745848
	0.5					0.232204
	0.7					0.271123
		0.7				0.232204
		0.9				0.288469
			0.2			0.232204
			0.4			1.62164
				0.7		0.232204
				0.9		0.295894
					0.01	0.232204
					0.02	0.279904

**Table 3:** Numerical simulations for $Nu_{Z} \left( {Re_{Z} } \right)^{{ - \tfrac{1}{2}}}$.

$Ec$	$\gamma_{3}$	$M$	$Nb$	$Nt$	$\Pr$	$Rd$	$Nu_{z} \left( {Re_{z} } \right)^{{ - \tfrac{1}{2}}}$
0.5	0.7	0.7	0.5	0.5	1.5	1.0	0.692796
0.6							0.671870
0.7							0.650944
	0.7						0.692796
	0.9						0.773657
		0.7					0.692796
		0.8					0.677195
			0.5				0.692796
			0.6				0.65948
				0.5			0.692796
				0.6			0.665348
					1.5		0.692796
					1.6		0.677874
						1.0	0.692796
						1.1	0.72645

**Table 4:** Numerical simulations for $Sh_{z} \left( {Re_{z} } \right)^{{ - \tfrac{1}{2}}}$.

$\delta_{1}$	$E_{1}$	$\gamma_{3}$	$\gamma$	$n$	$Nb$	$Nt$	$Sc$	$Sh_{z} \left( {Re_{z} } \right)^{{ - \tfrac{1}{2}}}$
0.6	0.5	0.7	0.5					0.665558
0.7								0.682322
0.8								0.698943
	0.5							0.665558
	0.8							0.619483
		0.7						0.665558
		0.9						0.66453
			0.5					0.665558
			0.7					0.758014
				1.0				0.665558
				2.0				0.773672
					0.5			0.665558
					0.6			0.678996
						0.5		0.665558
						0.6		0.680346
							0.6	0.665558
							0.8	0.766182

## Conclusions

The prime objective of this article is to examine the irreversibility in steady magnetized flow of Sutterby nanofluid caused by stretched cylinder. The features of Arrhenius kinetics, Joule heating, chemical reaction, Darcy Forchheimer, surface permeability and thermal radiation are accounted in development of mathematical governing equations. Through thermodynamics 2^nd^ law total irreversibility is modeled. Numerical and graphical solutions are constructed through RKF-45 in Mathematica package. Main findings are itemized as:

- Velocity decreases for higher porosity parameter and magnetic variable.
- Temperature have opposite behavior for magnetic variable and Prandtl number.
- For higher thermophoresis and Brownian movement variable temperature enhances.
- Concentration decreases for Brownian movement variable while it increases for thermophoresis variable.
- For higher Schmidt number concentration decreases.
- EG increases for higher Brinkman number, temperature difference ratio variable and magnetic variable.
- Bejan number decays versus higher Brinkman number and magnetic variable while it enhances for temperature difference ratio variable.

## Data Availability

All data generated or analyzed during this study are included in this published article.

## List of symbols

$m$: Power law index $\left( - \right)$
$$k$$: Thermal conductivity $$\left( {{\text{W}}\;{\text{m}}^{ - 1} {\text{K}}^{ - 1} } \right)$$
$\tau$: Heat capacity ratio $\left( - \right)$
$B_{0}$: Strength of magnetic field $\left( {{\text{kg}}\,{\text{s}}^{ - 2} {\text{A}}^{ - 1} } \right)$
$$\mu$$: Fluid friction $$\left( {{\text{kg}}\;{\text{m}}^{ - 1} {\text{s}}^{ - 1} } \right)$$
$$D_{T}$$: Thermophoresis dispersion $$\left( {{\text{m}}^{2} \;{\text{s}}^{ - 1} } \right)$$
$\rho$: Density of fluid $\left( {{\text{kg}}\;{\text{m}}^{ - 3} } \right)$
$$\left( {u,\;w} \right)$$: Velocity components $$\left( {{\text{ms}}^{ - 1} } \right)$$
$$T$$: Temperature $$\left( {\text{K}} \right)$$
$\sigma^{ * }$: Stefan Boltzmann constant $\left( {{\text{J}}\;{\text{s}}^{ - 1} {\text{m}}^{ - 2} \,{\text{K}}^{ - 4} } \right)$
$$C_{\infty }$$: Ambient concentration $$\left( - \right)$$
$k_{r}^{2}$: Chemical reaction rate $\left( {{\text{s}}^{ - 1} } \right)$
$D_{B}$: Brownian motion coefficient $\left( {{\text{m}}^{2} \;{\text{s}}^{ - 1} } \right)$
$$\tau_{w}$$: Shear stress $$\left( {{\text{Wm}}^{ - 2} } \right)$$
$$J_{w}$$: Mass flux $$\left( {{\text{ms}}^{ - 1} } \right)$$
$$\theta (\eta )$$: Temperature $$\left( - \right)$$
$R$: Radius of cylinder $\left( {\text{m}} \right)$
$$\Pr$$: Prandtl variable $$\left( - \right)$$
$$\lambda$$: Porosity parameter $$\left( - \right)$$
$$Re$$: Reynolds number $$\left( - \right)$$
$$Rd$$: Radiation parameter $$\left( - \right)$$
$Nt$: Thermophoretic diffusion variable $\left( - \right)$
$\delta_{1}$: Temperature difference ratio parameter $\left( - \right)$
$\gamma$: Rate of chemical reaction $\left( - \right)$
$$\nu$$: Kinematic viscosity $$\left( {{\text{m}}^{2} \;{\text{s}}^{ - 1} } \right)$$
$$B$$: Characteristic time $$\left( {\text{s}} \right)$$
$$\sigma$$: Electric conductivity $$\left( {{\text{kg}}^{ - 1} {\text{m}}^{ - 3} {\text{s}}^{3} {\text{A}}^{2} } \right)$$
$k_{p}$: Porous medium permeability $\left( {{\text{m}}^{2} } \right)$
$$F_{e}$$: Inertial coefficient $$\left( {{\text{kgm}}^{2} } \right)$$
$k^{ * }$: Coefficient of mean absorption $\left( {{\text{m}}^{ - 1} } \right)$
$$D_{B}$$: Brownian movement $$\left( {{\text{m}}^{2} \;{\text{s}}^{ - 1} } \right)$$
$$Ea$$: Activation energy $$\left( {{\text{kg}}\,{\text{m}}^{2} \,{\text{s}}^{ - 2} } \right)$$
$$C_{p}$$: Specific heat $$\left( {{\text{J}}\;{\text{kg}}^{ - 1} {\text{K}}^{ - 1} } \right)$$
$n_{1}$: Fitted rate constant $\left( - \right)$
$$\left( {r,\,z} \right)$$: Coordinate axes $$\left( {\text{m}} \right)$$
$$T_{\infty }$$: Ambient temperature $$\left( {\text{K}} \right)$$
$$C$$: Concentration $$\left( - \right)$$
$$q_{w}$$: Heat flux $$\left( {{\text{J}}\;{\text{s}}^{ - 2} } \right)$$
$$f^{\prime}\left( \eta \right)$$: Velocity
$$\phi (\eta )$$: Concentration $$\left( - \right)$$
$$\gamma_{3}$$: Curvature variable $$\left( - \right)$$
$$M$$: Magnetic variable $$\left( - \right)$$
$\beta_{1}$: Sutterby fluid parameter $\left( - \right)$
$$Fr$$: Forchheimer variable $$\left( - \right)$$
$Nb$: Brownian dispersion variable $\left( - \right)$
$E_{1}$: Activation energy parameter $\left( - \right)$
$$Ec$$: Eckert number $$\left( - \right)$$
$$Sc$$: Schmidt number $$\left( - \right)$$
